# Salmon bias effect as hypothesis of the lower mortality rates among immigrants in Italy

**DOI:** 10.1038/s41598-021-87522-2

**Published:** 2021-04-13

**Authors:** Anteo Di Napoli, Alessandra Rossi, Gianfranco Alicandro, Martina Ventura, Luisa Frova, Alessio Petrelli

**Affiliations:** 1 National Institute for Health, Migration and Poverty (INMP), Istituto Nazionale per la promozione della salute delle popolazioni Migranti e per il contrasto delle malattie della Povertà (INMP), Via di S. Gallicano 25/a, 00153 Roma, Italy; 2 National Institute of Statistics (Istat), Viale Liegi 13, 00198 Rome, Italy; 3 Department of Pathophysiology and Transplantation, Università degli Studi di Milano, Milan, Italy

**Keywords:** Epidemiology, Public health

## Abstract

Compared with natives, immigrants have lower all-cause mortality rates, despite their lower socioeconomic status, an epidemiological paradox generally explained by the healthy migrant effect. Another hypothesis is the so-called salmon bias effect: “statistically immortal” subjects return to their country of origin when they expect to die shortly, but their deaths are not registered in the statistics of the country of residence. This underestimation of deaths determines an artificially low immigrant mortality rate. We aimed to estimate the potential salmon bias effect on differences in mortality rates between Italians and immigrants. We used a national cohort of all Italians registered in the 2011 census and followed up for mortality from 2012 to 2016. Mortality data were retrieved from the Causes of Death Register, which included all deaths occurring in the country and the Resident Population Register, which collects also the deaths occurring abroad. We assumed as a possible salmon bias event the death of an immigrant resident in Italy that died in his/her country of origin. Considering the deaths occurring in the country of origin, we observed an 18.1% increase in the overall mortality rates for immigrants and an increase of 23.7% in the age-standardized mortality rate. Mortality rates of immigrants resident in Italy, calculated without taking into account the deaths occurring in the country of origin, are certainly underestimated. However, the salmon bias only partly explains the difference in mortality rates between immigrants and Italians.

## Introduction

Compared with natives, immigrants generally have a lower all-cause mortality rate despite their lower socioeconomic status, which is associated with poor health in terms of both morbidity and mortality^1,2^. Several explanations have been proposed for this epidemiological paradox.

The healthy migrant effect hypothesis posits that migration is selective of healthier individuals: migrants are healthier than the native people both of the country of origin and of the country of destination^1,3–5^.

A second hypothesis is the so-called salmon bias effect, an expression first used by Pablos-Mendez to describe “the compulsion to die in one’s birthplace”^6^. This hypothesis asserts that many immigrants return to their country of origin when they expect to die shortly^1,5–7^. If deaths occurring in their country of origin are not registered in the mortality statistics of the country of residence, some individuals become “statistically immortal”, resulting in an artificially low immigrant mortality rate^1,3,5,6^. The salmon bias was advanced as a possible explanation of the “Hispanic paradox”, the lower mortality rate of Hispanics than of non-Hispanic whites in the United States, despite the former having a more disadvantaged risk factor profile^1,3,5,6^.

Several studies have evaluated the hypothesis that any survival advantage for immigrants compared to natives may merely be a statistical artifact; the mobility of immigrant populations may cause an undercoverage of deaths and/or an overcoverage of the resident population in demographic registers^8–12^.

However, the salmon bias has not been convincingly documented^7^, and, to our knowledge, it has never been evaluated in Italy. Previous Italian studies found that immigrants showed a lower risk of mortality compared to Italians^2,13^, although unregistered remigration (delays in registration in municipal registries of the final return to the country of origin), which inflates the mortality rate denominators, has been postulated^13^.

We aimed to estimate the potential salmon bias effect on differences in mortality rates between Italians and immigrants resident in Italy.

## Methods

The study was conducted using the Italian statistical registers, which are the main source of demographic statistics in our country. In particular, we used the 15th Census of Population and Housing (2011), the Causes of Death (CoD) Register, and the Resident Population (RP) Register. The RP collects individual data on demographic events occurring in Italy or abroad among the resident population, such as births, deaths, and migrations.

The study was based on a national cohort made up of all residents recorded in the 2011 Census with follow-up data for mortality from January 2012 up to December 2016^14^. Subjects entered the cohort on 1 January 2012 and were followed up until death, emigration, or last available year of mortality data (2016), whichever came first, yielding a maximum of 5 years of follow-up. Mortality data were obtained through a deterministic record linkage with the CoD Register by using the fiscal code (a unique personal identification number issued to all residents in Italy at birth or upon immigration) as linkage key. The reliability of the fiscal code was very high in all the registers, making it possible to link 97.1% of all deaths among the Census population occurring in Italy in the period 2012–2014^15^. Since there is no reason to believe that reliability of the fiscal code reported in all registers decreased over the subsequent years, the performance of the record linkage is expected to be equally high.

The CoD Register annually collects information on deaths occurring in Italy among the resident population but does not record deaths occurring abroad. To recover those deaths, we also linked the census archive and the RP Register with a deterministic procedure, using the fiscal code as linkage key. The RP Register was also used to recover the date of migration for those who had moved abroad.

The study used Istat official registers, which were checked for duplications by the Institute itself before the final release. The post-enumeration survey estimated an undercoverage rate of 1.07% for the 2011 Italian Census^16^; missing deaths are unlikely since mortality data in Italy cover 100% of the population^17^.

We assumed as a possible salmon bias all events occurring among immigrants who were resident in Italy on the date of the census, who died in their country of origin, and who were then not recorded in the Causes of Death Register.

The country of origin was identified through citizenship for two reasons: first, in Italy, where the phenomenon of migration is relatively recent, citizenship represents the status of immigrants better than does the country of birth. Italian citizenship is acquired by foreign adults only after long, continuous residence or by children of foreigners born in Italy when they turn 18. Second, the information about country of birth was affected by too many missing values.

We calculated crude, age-specific and age-adjusted mortality rates, and the ratio between the age-adjusted mortality rate computed with and without the deaths occurring in the country of origin. Age-standardized mortality rates were computed using European population in 2013 as standard.

The cohort was conceived within the project “IF IST 2646 ‐ Socioeconomic differences in mortality”, which was included in the National Statistical Program and approved by the Italian Data Protection Authority.

## Results

The study cohort included 59,227,313 individuals (55,221,311 Italians and 4,006,002 immigrants). Immigrants were younger than Italians (median age class: 30–34 vs. 40–44), while the percentage of males was lower among immigrants than Italians (46.7% vs. 48.5%).

In the period 2012–2016, there were 17,158 deaths among immigrants occurring in Italy and another 3,102 deaths occurring in the country of origin, accounting for an 18.1% increase in detected deaths. Correspondingly, among immigrants, the crude mortality rate increased from 8.70 to 10.27 deaths per 10,000 person-years, while the standardized mortality rate increased from 38.19 to 47.23 deaths per 10,000 person-years.

Deaths of immigrants from Albania (N = 879), Morocco (N = 467), and Romania (N = 385), the three most numerous foreign communities in Italy (41.8% of the immigrant population), accounted for 55.8% of all deaths occurring in the country of origin. The detailed mortality rates for the 20 countries with the highest number of deaths are shown in Table 1.

**Table 1:** Total deaths, crude and age-standardized mortality rates (ASMRs) (per 10,000 person-years) among immigrants resident in Italy, calculated with and without the deaths occurred in the country of origin, by country.

Country	Total Deaths (n.)	Deaths in the country of origin (n., %)		Crude mortality rate (excluding the deaths occurred in the country of origin)	Crude mortality rate (including the deaths occurred in the country of origin)	ASMR (excluding the deaths occurred in the country of origin)	ASMR (including the deaths occurred in the country of origin)
Romania	3040	385	12.7	6.6	7.5	42.3	54.8
Albania	3031	879	29.0	9.6	13.6	30.7	48.5
Morocco	1662	467	28.1	6	8.3	16.1	28.3
Germany	1008	111	11.0	55.1	62	51.6	58.4
Ukraine	732	80	10.9	7.4	8.3	24.1	26.2
France	602	43	7.1	50.2	54.1	56.7	61.8
Philippines	546	39	7.1	8	8.6	18.1	22.4
United Kingdom	536	16	3.0	49	50.5	55.5	57.4
India	482	53	11.0	7.6	8.5	45.3	50
Switzerland	444	24	5.4	113.5	120.2	65.7	69.9
Poland	431	44	10.2	9.4	10.5	35.4	40.5
China	420	25	6.0	4.1	4.4	24.5	25.6
Moldova	409	65	15.9	5.4	6.4	34	39.7
Macedonia	330	104	31.5	6.3	9.2	31.1	61.2
Serbia and Montenegro	326	56	17.2	12	14.5	52.4	68.3
United States of America	312	11	3.5	53.2	55.1	48.6	50.4
Peru	308	32	10.4	6	6.7	22.1	25.1
Senegal	279	56	20.1	6.2	7.8	18.6	23.2
Tunisia	265	53	20.0	5.2	6.6	17.1	25.5
Ghana	242	30	12.4	9.8	11.2	22.9	28.7

Data are shown only for the 20 countries with the highest number of deaths registered in the period 2012–2016.

**Figure 1:**
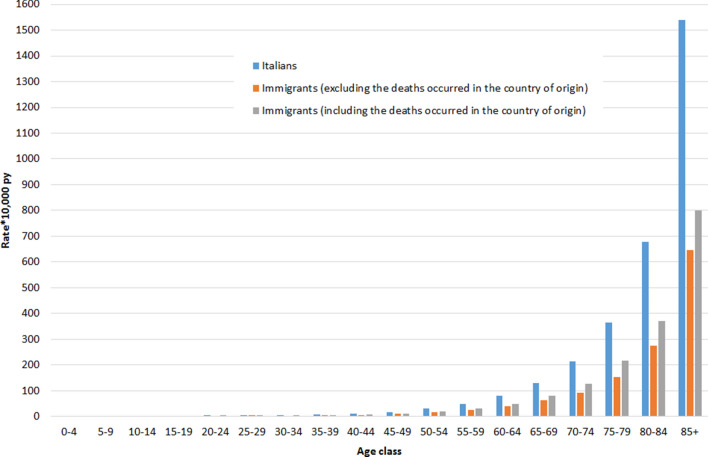
Age-specific mortality rates*10,000 person years among men resident in Italy, by citizenship and source of data. Istat, 2012–2016.

**Figure 2:**
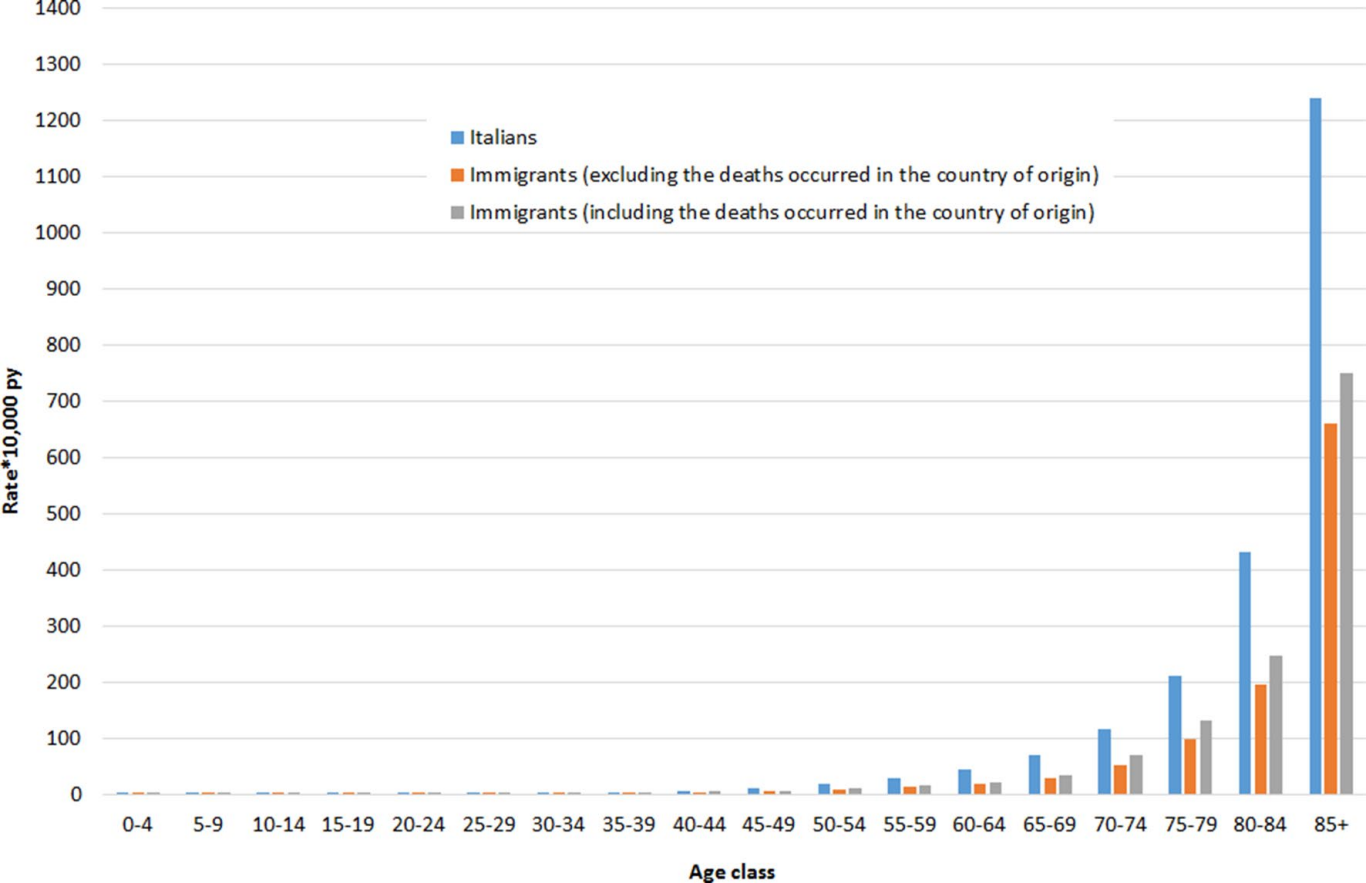
Age-specific mortality rates*10,000 person years among women resident in Italy, by citizenship and source of data. Istat, 2012–2016.

Figures 1 and 2 shows the age-specific mortality rates for Italians and for immigrants, calculated with and without deaths occurring in the country of origin, for men and women, respectively.

The crude mortality rate at all ages per 10,000 person-years calculated without the deaths occurring in the country of origin was: for Italians, 106.37 (95% CI: 106.31–106.42) for men and 108.11 (95% CI: 108.05–108.16) for women; for immigrants, 10.00 (95% CI: 9.93–10.06) for men and 7.56 (95% CI: 7.51–7.61) for women. Instead, when considering also the deaths occurring in the country of origin, it was 11.91 (95% CI: 11.84–11.98) for men and 8.84 (95% CI: 8.78–8.89) for women.

After age standardization, the mortality rate per 10,000 person-years decreased to 101.75 (95% CI: 101.58–101.92) and 67.70 (95% CI: 67.59–67.81) for Italian men and women, respectively. For immigrants, the age-standardized rates, calculated without the deaths occurring in the country of origin, were 45.06 (95% CI: 43.66–46.51) for men and 33.64 (95% CI: 33.62–34.70) for women. Instead, when also considering the deaths occurring in the country of origin, these rates increased to 57.69 (95% CI: 56.41–58.96) for men and 40.40 (95% CI: 39.46–41.34) for women.

**Figure 3:**
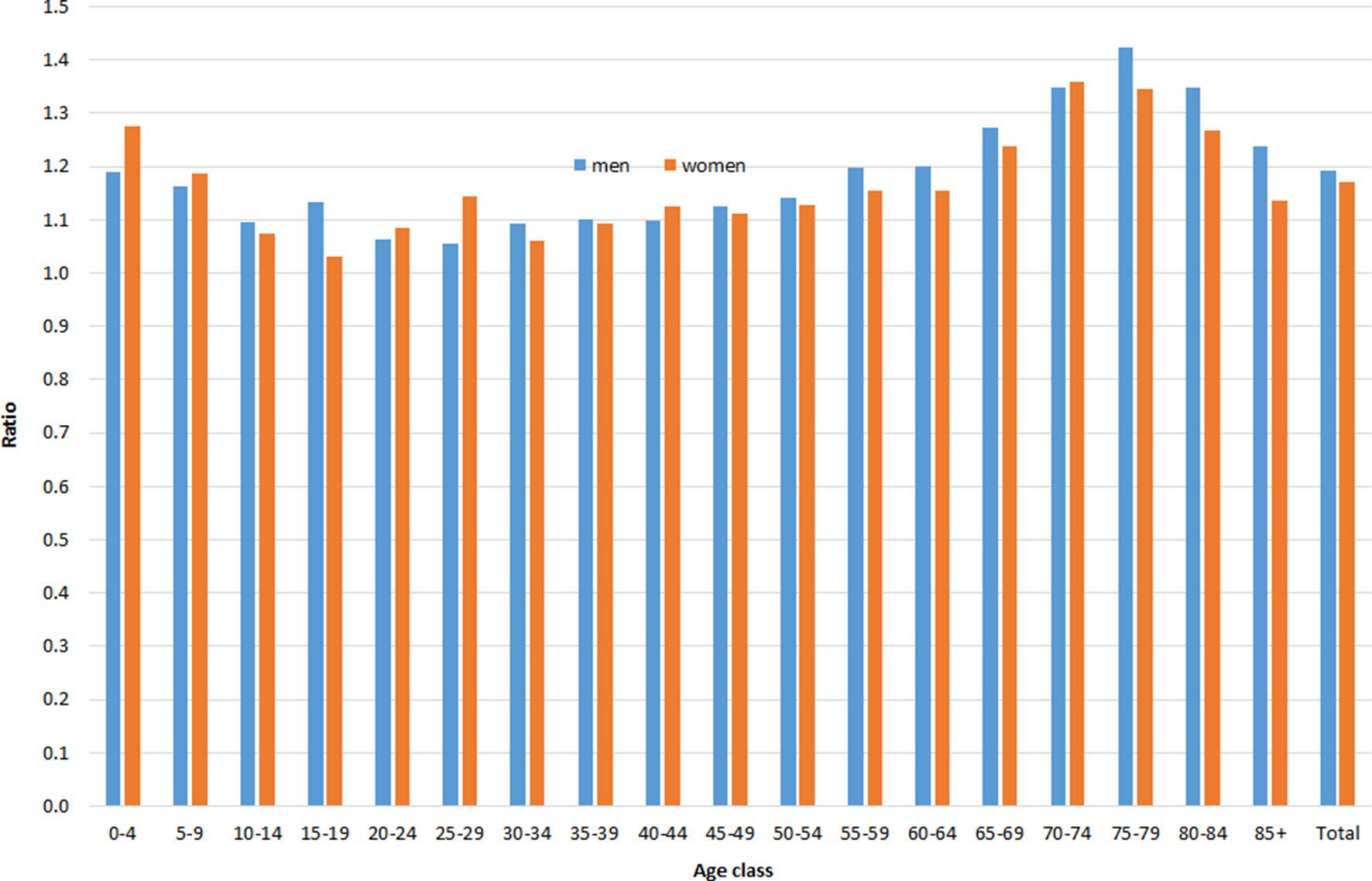
Ratio between age-specific mortality rates of immigrants resident in Italy, calculated including and excluding deaths occurred in the country of origin, by sex. Istat, 2012–2016.

Figure 3 shows the ratio of the age-specific mortality rates among immigrants, calculated with and without deaths occurring in the country of origin, with an average excess of about 19% for men and 17% for women. The ratio was particularly high for subjects aged 65–69 (1.27 for men and 1.24 for women), 70–74 (1.35 for men and 1.36 for women), and 75–79 (1.42 for men and 1.34 for women). Similar patterns in ratios were found for subjects aged ≥ 65 when focusing the analyses on the three most numerous foreign communities in Italy: the Romanian (men: 1.40, women: 1.29), Albanian (men: 1.77, women: 1.29) and Moroccan (men: 1.84, women: 1.96) communities (data not shown in figure).

## Discussion

In our study we observed that considering the deaths occurring in the country of origin increased the overall mortality rates of immigrants resident in Italy by 18.1% and the age-standardized mortality rate by 23.7%.

Indeed, the age-standardized mortality rates for immigrants computed without the deaths occurring in the country of origin were much lower than those for Italians and remained lower even when we considered the deaths occurring in the country of origin.

Thus, it can be said that studies based only on the CoD Register in the country of residence underestimate the mortality rates of immigrants resident in Italy.

The estimation of mortality among the immigrant population is subject to a number of data problems due to the limits in recording mobility among foreign-born populations^8,11^. National vital registration systems are poorly equipped to gather information on residents dying abroad, in particular if they have foreign citizenship. The problem concerning data mismatch arises when deaths occur without an official residence change, so that person-years are still accounted for in national mortality rate estimates^11^.

The detection of “statistically immortal” subjects could fall within the definition of the salmon bias, which refers to those deaths occurring in the country of origin but not registered in Civil Registry Office of the country of residence. This results in an underestimation of the numerator and, consequently, an artificially low immigrant mortality rate^1,3,5–12^.

Another point to support the presence of the salmon bias is that we found the greatest differences between the two methods for calculating the deaths of immigrants among the elderly, supporting the hypothesis that if deteriorating health triggers return migration, this phenomenon is more pronounced at older ages, when health problems become more prevalent^5,7,13^. These differences are confirmed by our findings within the three largest foreign communities in Italy (Romania, Albania, Morocco). Some authors have described this as a differential remigration by age, which can affect especially those immigrants with health problems and/or low socioeconomic profiles (unhealthy remigration effect)^13^.

Once having emigrated, individuals are less likely to have their vital events documented in their country of origin, where they are still registered and continue to be erroneously regarded as being at risk of vital events. This represents a potential source of bias in register-based censuses, defined as overcoverage, which has been postulated as one of the explanations of the migrant mortality paradox, in particular at peak migration ages^8,9,11^.

Among immigrants, mortality generally shows a U-shaped age pattern, with excess at young and older ages compared to natives, with a large advantage at adult ages^8^. The problem of the reliability of very low estimates of migrant mortality at ages 65 and over has been raised, given the risk of immigrants’ potentially not having un-registered from host country registries^2,12^. In our study we observed the largest differences in mortality rates between immigrants and Italians at older ages.

However, the retrieved information on the unregistered deaths of immigrants, even if we hypothesize the presence of the salmon bias effect, is not enough to explain the large difference in mortality rates compared to those of Italians. In fact, the age-specific and standardized mortality rates of immigrants remain much lower than those observed among Italians, even with the addition of the deaths detected by the new source.

The salmon bias effect may contribute to explain the observed difference in mortality rates between Italians and immigrants; the role of the healthy migrant effect remains prominent, as observed also by other studies that tested the hypothesis of potential statistical artifact^8,9^.

The better health status of the immigrant population compared to that of Italians could be ascribed to the well-known positive selection effect: healthier people are more likely to migrate^2,10,13^.

An interesting research hypothesis that could confirm the effect of the salmon bias is to verify the increase in mortality rates of those immigrants who have returned compared to those of the general population of their country of origin. In fact, if the low mortality among immigrants compared to that of the country of destination is partly explained by the salmon bias, we would expect increased mortality among repatriates compared with the population in the country of origin, in particular in the first few years after repatriation^7,10^. Such a study could be conducted, for example, also on the population of Italian emigrants who have returned from abroad over the past few decades.

The study has some limitations.

First, our findings represent only indirect empirical evidence that could either refute or support the salmon bias hypothesis as an explanation for the lower mortality observed among immigrants compared to Italians. This evidence is thus probably insufficient to confirm the salmon bias hypothesis.

Second, we do not know the causes of death of individuals who died in their country of origin since this crucial information is not collected in the RP Register. In fact, in the presence of the salmon bias, one would expect higher mortality rates for some specific causes, for example, cancer, especially immediately after returning to one’s country of origin^10^. Moreover, it was not possible to determine whether immigrants died in their country of origin of acute causes, such as infectious diseases or accidents, but this would not in any case contribute to the salmon bias hypothesis.

Third, due to the characteristics of the data source used to collect deaths occurring abroad, the date of the return to the country of origin is unknown because for the RP Register, these individuals are still resident in Italy. The RP Register can only integrate the information about deaths of immigrants undetected by the routine CoD Register source.

Finally, we do not know whether all immigrants who die in their country of origin are captured in RP Register. In any case, this limitation would not conflict with the hypothesis of the presence of a salmon bias effect.

To our knowledge, this is the first study that has attempted to estimate whether the salmon bias effect has any role in explaining the lower mortality of immigrants in Italy than that of Italians. Another strength of the study is the integration of two complete and reliable national archives, the CoD Register and the RP Register.

## Conclusions

The mortality rates of immigrants resident in Italy, calculated so far without taking into account the deaths of subjects who died abroad, are certainly underestimated. Future studies on the mortality of immigrants in Italy will necessarily have to take this into account. The lower mortality rate among immigrants compared to that of natives is a real phenomenon, but researchers must take into account the potential biases when they estimate the extent of the advantage.
